# AMTAS^TM^ and user-operated smartphone research application audiometry—An evaluation study

**DOI:** 10.1371/journal.pone.0291412

**Published:** 2023-09-14

**Authors:** Chris Bang Sørensen, Thomas Bording Adams, Ellen Raben Pedersen, Jacob Nielsen, Jesper Hvass Schmidt

**Affiliations:** 1 The Maersk Mc-Kinney Møller Institute, Faculty of Engineering, University of Southern Denmark, Odense, Denmark; 2 Research Unit for ORL–Head & Neck Surgery and Audiology, Odense University Hospital & University of Southern Denmark, Odense, Denmark; 3 OPEN, Odense Patient data Explorative Network, Odense University Hospital, Odense, Denmark; Universidad de Chile, CHILE

## Abstract

**Objective:**

To evaluate two user-operated audiometry methods, the AMTAS^TM^ PC-based audiometry and a low-cost smartphone audiometry research application (R-App).

**Design:**

A repeated-measures within-subject study design was used to compare both user-operated methods to traditional manual audiometry and to evaluate test-retest reliability of each method.

**Study sample:**

58 subjects were recruited in the study of which 83 ears had normal hearing thresholds and 33 ears had hearing loss (pure-tone average > 25 dB HL). Average age of participants was 44.8 years, with an age range of 11–85.

**Results:**

Standard deviation of absolute differences ranged between 3.9–6.9 dB on AMTAS^TM^ and 4.5–6.8 dB on the R-App. The highest variability was found at the 8000 Hz frequency (R-App and AMTAS^TM^ test) and 3000 Hz frequency (AMTAS^TM^ retest). Evaluation of test-retest reliability of AMTAS^TM^ and R-App showed SD of absolute differences ranging between 3.5–5.8 dB and 3.1–5.0 dB, respectively. The mean threshold difference between test and retest was within ±1.5 dB on AMTAS^TM^ and ±1 dB on the R-App.

**Conclusion:**

Accuracy of AMTAS^TM^ and the R-App was within acceptable limits for audiometry and comparable to traditional manual audiometry on all tested frequencies (250–8000 Hz). Evaluation of test-retest reliability showed acceptable variation on both AMTAS^TM^ and R-App. Both user-operated methods could be reliably performed in a quiet non-soundproofed environment.

## Introduction

Hearing loss is a progressive and escalating health concern, with an estimated 466 million people experiencing disabling hearing loss, thus affecting approximately 5.5% of the current world population [1]. Disabling hearing loss is defined for adults as a greater than 35 dB HL in the better hearing ear [1]. The number of people experiencing disabling hearing loss is projected to increase to over 700 million people by 2050 [2]. The increasing prevalence of hearing loss is purportedly attributed largely to three sources: a greater life-expectancy, noise exposure arising from occupational, environmental and recreational sources, and the use of ototoxic medication [3]. Consequently, more clinicians need to be trained and more need to remain in the field until retirement currently to meet future audiological demands [4]. Alternatively, technological advances in user-operated audiological equipment are expected to increase the accessibility of diagnostic audiometry, and their relevance were only further exacerbated by the Covid-19 pandemic [5].

A method for self-recording audiometry was proposed over 70 years ago by von Békésy. The Békésy audiometry continuously changes the intensity level of presented stimuli and uses either a sweeping or constant frequency (Békésy sweep or Békésy fixed frequency method). The continuously changing intensity level of the stimuli is kept merely audible by having the patient push a button when and for as long as they can hear the stimuli [6, 7]. Since then, many innovative new portable and user-operated audiometry systems have been tested, particularly in the era of smartphones and tablets, e.g., app-based solutions such as uHear^TM^ (Unitron, Commak, New York), Shoebox (Clearwater Clinical, Ottawa, Canada), EarTrumpet (Praxis Biosciences, Irvine California) and hearScreen^TM^ (hearX group, Pretoria, South Africa). Across several studies, the uHear^TM^ app produced elevated thresholds compared to the gold standard traditional manual audiometry, and suffered from low specificity when testing was performed in non-sound-treated settings, showing that environmental ambient disruption significantly affected the accuracy of the app [8–12]. In a newer study on the uHear^TM^ app performed in a non-sound-treated room, results were only elevated when using supra-aural headphones and comparable to manual audiometry when circumaural headphones (Bose QC3, earcup cushions) were used [13]. Results from the Shoebox app gave thresholds within 10 dB of those found with manual audiometry [14], findings that were comparable to measurements found within studies on the EarTrumpet app [15]. Both the Shoebox and EarTrumpet apps were developed for the iOS platform, limiting their reach to upper-end merchandise exclusive to Apple (Apple Inc., Cupertino, California). van Tonder, Swanepoel [16] evaluated the hearTest^TM^ app with an inexpensive Android device. The study revealed within-subject mean threshold differences between conventional and smartphone user-operated audiometry in the range -3.3 to 2.9 dB. A limitation of the equipment in the study was a floor effect at 10 dB HL, excluding all potential or viable thresholds pertaining to a lower limit of 10 dB HL [16].

Research methodologies used in user-operated audiometry have also been studied and evaluated on other portable equipment. One of these is the AMTAS^TM^ (Automated Method for Testing Auditory Sensitivity), a system with an external audiometer connected to a laptop, which includes both air- and bone-conducted threshold measurements for either screening or diagnostic purposes [17]. Features of AMTAS^TM^ include masking and patented quality control features [18]. Results from Eikelboom et al. [19] revealed standard deviation of absolute threshold differences between 5.9 and 6.9 dB, when air-conducted measurements were compared between AMTAS^TM^ and manual audiometry. Mean test-retest differences in air-conducted measurements ranged from -1.5 dB (250 Hz) to 1.8 dB (8000 Hz) with a SD of absolute differences range of 4.1–6.0 dB [19]. Notable differences between this and prior AMTAS^TM^ studies were purportedly attributable to environmental factors, such as a quiet ambience as opposed to the sound-treated domain used in the prior studies [17, 19].

In this paper we report the development and evaluation of a new low-cost smartphone application for detecting air-conducted thresholds (the “Research App”, R-App), written for the Android OS platform. Because of the inconsistency of findings in previous studies, we also reevaluated AMTAS^TM^ in a non-sound-treated setting to provide the field with more data on this method. The overall aim of this study was to determine the individual efficacy of the R-App and AMTAS^TM^ in environmental conditions, as an alternative to manual audiometry conducted in a sound-treated room. Our specific objectives were (1) to assess the accuracy of the R-App and AMTAS^TM^, i.e., compare air-conducted thresholds to manual audiometry, and (2) to determine the test-retest reliability of the user-operated solutions.

## Methods and materials

### Subjects

The study was approved by the local ethical committee, The Health Research-ethics Committees of Southern Denmark, and all subjects provided written informed consent prior to any data collection. Written informed parental consent was obtained for adolescents (subjects below 18 years of age). Adolescents were not a particular interest of this study but were not excluded if one came through the recruitment channels (two subjects were adolescents). Sixty-one subjects were recruited, with thirty being nearby residents or university students, and thirty-one being patients at Odense University Hospital (OUH)–either the Department of Audiology or the Department of Oncology. Three out of the sixty-one subjects could not complete the user-operated audiometries (due to exhaustion, lack of dexterity) and were later excluded, leaving fifty-eight subjects in the study. Prior to enrolment, the subjects were asked to declare any acute otological symptoms that could influence or circumvent the outcome of the user-operated audiometry and to reschedule their test if such manifestations were present at time of arrival. Only subjects with hearing thresholds below 80 dB HL at the octave frequencies 250–8000 Hz and inter-octave frequencies 3000 and 6000 Hz were included to ensure subject thresholds were comfortably within the dynamic range capability of the equipment. All subjects were native Danish speakers. See Table 1 for the demographic characteristics of the 58 subjects.

**Table 1:** Subject demographics.

Subjects, *n*	58	
Age (years) median and range	37.5	(11–85)
Mean age	44.8	
Distribution of age (years), *n*		
(11–20)	3	
(21–40)	26	
(41–60)	7	
(61–85)	22	
Sex, *n* (%)		
Female	26	(45%)
Male	32	(55%)
Ears with PTA (dB), *n*		
≤ 25 (normal hearing)	83	
26–40 (slight impairment)	31	
41–60 (moderate impairment)	2	
> 60 (severe impairment)	0	

Subject demographics and hearing loss. Pure-tone averages (PTAs) were calculated based on the manual audiometry thresholds obtained during the study.

### Gold standard traditional manual audiometry

All subjects had their hearing measured by audiologists using standard manual audiometry in sound-treated rooms at the Department of Audiology, OUH. Subjects with known hearing loss [27] had been measured during the six months before enlisting in the study. Subjects without known hearing loss [31] were tested once by audiologists at the same facilities after enrolment. All subjects were examined bilaterally with otoscopy by the audiologist prior to testing in order to detect cerumen blockage of the external ear canal that potentially could influence the testing results. Pure-tone air-conducted thresholds at octave (250 to 8000 Hz) and inter-octave (3000 and 6000Hz) frequencies were examined. The manual audiometry was carried out using a MADSEN Astera2 audiometer (Otometrics Natus Medical Denmark) with Otometrics insert headphones and in accordance with ISO 8253–1:2010 standards for audiometric procedures. During examination, the audiologist used pure-tone or alternatively warble tones if deemed necessary for adequate threshold determination. Pure-tone thresholds were recorded in the database for each patient and compared to test and retest results from R-App and AMTAS^TM^.

### AMTAS^TM^ PC-based user-operated audiometry

AMTAS^TM^ is a system designed for auditory threshold testing via a PC. AMTAS^TM^ uses a forced choice algorithm with “YES” or “NO” options after stimulus presentation, thereby differentiating itself from manual audiometry where a response is required only when the patient has heard the stimuli. The stimuli presented were pure-tones only. AMTAS starts at 40 dB HL and initially follows 10 dB-down 10 dB-up algorithm before becoming 10 dB-down 5 dB-up. AMTAS^TM^ is further described in its patent [20] and first two publications [17, 18]. The setup used was a combination of an external Calisto^TM^ audiometer (Interacoustics, Middelfart, Denmark) delivering the sound output connected to a touch-screen portable 3^rd^ generation ThinkPad X1 Yoga Lenovo (Lenovo Group, Quarry Bay, Hong Kong) computer via a USB connection. Connected to the Calisto^TM^ was a pair of RadioEar DD450 circum-aural headphones, the same model used with the smartphone and R-App setup. Eikelboom et al. [19] describe these headphones as suitable for use in non-sound-treated environments, due to their noise attenuation profile. AMTAS^TM^ featured two options: screening and diagnostic testing. The diagnostic option was chosen for testing the same eight frequencies as manual audiometry (250–8000 Hz including inter-octave frequencies 3000 and 6000 Hz) and with masking performed by the AMTAS^TM^ system. An advantage of the AMTAS^TM^ system is the quality indicators provided with the audiogram, which would be used to determine if an audiogram was not sufficiently trustworthy.

### Research App (R-App) user-operated audiometry

The R-App was designed at the Maersk McKinney Moeller Institute, SDU Odense. It uses a simple 10 dB-down 5 dB-up algorithm, starting the stimuli at 40 dB HL at each frequency. The R-app also uses a forced choice paradigm with “YES” or “NO” options after stimulus presentation. Stimuli are presented at the administered dB level for one second, with half a second of “ramp-up” and “ramp-down” on each side, for a total of two seconds of stimulus exposure. The R-App was developed for testing hearing thresholds at eight frequencies (250–8000 Hz), starting at 250 Hz on the right ear, followed by the left ear at the same frequency and continuing in this manner throughout the test until thresholds has been determined for both ears at all eight frequencies. A threshold is recognized when the subject answers “YES” to an applied intensity three times at the given frequency. A feature not currently in the R-app is masking, which might be an issue with asymmetrical hearing losses.

### Smartphone and sound-output equipment

**Fig 1:**
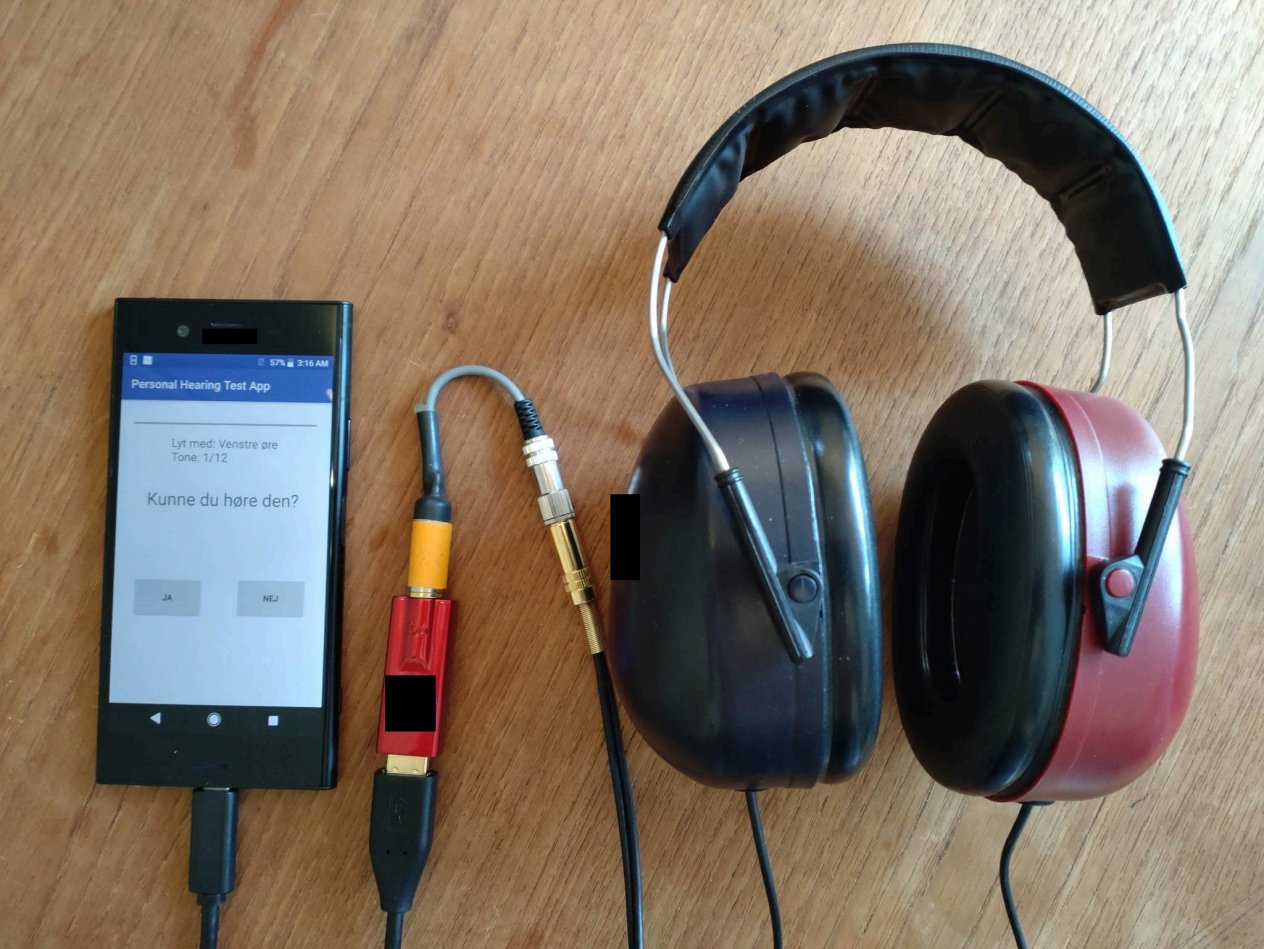
The R-App and smartphone equipment. From left to right: A Sony Xperia XZ1 smartphone. The smartphone is connected to a Dragonfly Red Digital to Analog Converter (DAC). The DAC is connected to a 400-ohm resistance, which lowers both the floor and ceiling of the systems output. Connected to the 400-ohm resistance is a pair of DD450 headphones. The version of the R-App pictured is a newer iteration where more information was added to the UI.

The R-App was installed on a Sony Xperia XZ1 smartphone (Sony Group Corp, Tokyo, Japan) via a USB type-C cable connection. Connected to the phone was an Android-compatible Dragonfly Red (Audioquest, Irvine, California) digital to analog converter (DAC). A DAC is used to bypass the smartphone auditory circuitry and deliver a wider dynamic range. Connected to the DAC was a pair of RadioEar DD450 circum-aural headphones (Interacoustics, Middelfart, Denmark) in series with a 400-ohm resistance, as Seen in Fig 1.

This setup was calibrated using a type 4153 artificial ear (IEC 60318–1 coupler), Nexus conditioning amplifier, sound calibrator and preamp (Brüel&Kjær, Nærum, Denmark) at FORCE Technology facilities, Odense, Denmark, prior to the enrolment. The calibration determined the maximum output of the full system for each pure-tone test frequency. These values were then used by the R-App in the equation 10^(*dBIntended^−^dBFrequencyMax*)/20^ = *volum*e, to set the volume of the android smartphone to achieve the intended dB of the stimuli. The resistor was added to offset the dynamic range of the headphones. This was done partly to prevent the intensity level exceeding 100 dB Sound Pressure Level (SPL) on any tested frequencies, and partly to prevent a floor effect at 10 dB SPL at 250 and 500 Hz. With the described equipment, the R-App could produce a max output of 96.8 dB SPL at 8000 Hz. This corresponded to 79.4 dB HL after conversion from dB SPL to dB HL (ISO 389–8:2004) and was the lowest maximum HL intensity of the R-App’s frequencies. All frequencies were tested for linearity down to 5 dB SPL, where ambient noise interfered with further measurements. The theoretical dynamic range of the system was calculated using the Signal-to-Quantization-Noise Ratio [21]. With the installed version of Android OS (Android 8) supporting up to 16 bit depth, the highest theoretical floor effect was calculated to be -4.3 dB HL at 1000 Hz and the lowest was -18.7 dB HL at 8000 Hz. The dynamic ranges were -10 to 85 dB at 250 and 4000 Hz, 0 to 90 dB at 500 and 1000 Hz, -5 to 90 dB at 2000 and 3000 Hz, and -15 to 80 dB at 6000 and 8000 Hz.

### Test conditions

AMTAS^TM^ and R-App were both tested in a non-sound-treated consultation room at the Department of Audiology, OUH. The consultation room was located off the clinics’ main hallway with light to moderate patient traffic and little external interruption. Ambient noise in the consultation room was measured during 10 minutes with one measurement point made per second for a total of 600 measurement points. Measurement points were SPL averages consisting of 1 second of recording. The equipment and study of multiple rooms (including the one in this study) are described in [22]. Only 14 measurement points at 250 Hz and 2 measurement points at 500 Hz were outside limits of ISO 8253–1:2010 with the attenuation of the RadioEar DD450 added [22]. Once seated in the office, the subjects would receive a brief oral presentation outlining the two user-operated methods. Following this, they received a video presentation accessible from the R-App home screen, and a modified version of the AMTAS^TM^ instructional video with a voice-over in Danish. After the presentation, subjects were prompted to begin, which they did by placing the headphones in the advised position and starting the test. A researcher from the team conducting the study was present throughout to assist in clarifying instructions or detail if needed by the subjects. The order of testing between the two methods was altered and counterbalanced between subjects, ensuring that subjects would begin with opposite systems upon test and retest. If the R-App was the first method to be tested, AMTAS^TM^ would then be the first method to be tested on retest. The subject would receive a 10-minute break inside the consultation room before a retest of the two user-operated methods took place. Each system was tested twice for test-retest reliability analysis, and pure-tone thresholds for each frequency were compared to those of manual audiometry.

### Data collection

All data was collected and stored in the online Research Electronic Data Capture (REDCap) tools developed by Vanderbilt University, Nashville, Tennessee, United States [23, 24], supplied by Odense Patient Data Explorative Network (OPEN). All data collected in REDCap was stored on OPEN’s servers in the Region of Southern Denmark with online access granted to project researchers by a data manager at the OPEN facility quarters. All online activity was logged on the OPEN servers and supervised by the data manager. Before enrolment, all subjects were enlisted in the database with a personal profile, generating a personal identification-number (ID) for that individual only. The R-App did not store any data on the local storage unit, instead, when a test was completed, results were sent directly to the REDCap database and stored in the personal profile corresponding to the subject’s generated ID. AMTAS^TM^ results were displayed graphically on the screen and saved locally until they were typed into the REDCap database manually after each subject’s participation. Likewise, manual audiometry results were printed by the performing audiologist and handed to a member from the research team and typed into the database manually.

### Statistical approach

It is assumed, that a difference in measured hearing thresholds between test-methods should be within +/-5 dB to be acceptable. Furthermore, the standard deviation of differences between repeated tests of the same type should ideally not exceed 5 dB. Further, it is assumed that each test has equal standard deviation of differences of 5 dB between test and retest, then a sample of 26 subjects in each group is sufficient to demonstrate that with a power of 0.9 and a significance level of 5%. The sample size of the study is robust enough to allow a standard deviation of difference up to 8 dB. This will require 54 subjects in each group.

Comparison between user-operated methods and manual audiometry was conducted by calculating means and standard deviations (SD) of real and absolute threshold differences for each tested frequency. Mixed-effects multilevel regression was also applied to test for statistical significance of any difference between tests. Thresholds were the response with ear and test as the fixed effect variables and person as the random effect variable. Bonferroni-Holm correction was done to account for the multiple statistical comparisons [25]. Bland-Altman plots [26] were used to visualize differences in measurements between manual audiometry and each of the user-operated audiometry tests. The limits of agreement method used by Bland & Altman provides an alternative to correlation analysis as it includes the mean differences between quantitative measurements of the two compared methods [27]. The selective plotting of the frequencies with the lowest and highest variability visualizes the spread in differences and further indicates whether there is proportional bias in the data, which would indicate that test variability was dependent on the level of the hearing thresholds. Test-retest reliability for each user-operated method was evaluated with the same approach. Combined standard uncertainty and expanded measurement uncertainty was calculated in accordance with ISO 8253–1:2010 (Annex A) for the audiometric uncertainties.

### Missing or invalid data

For the fifty-eight subjects included in the analysis, some data points were either missing, wrong, or deemed of poor quality by the AMTAS^TM^ method. These data points are all detailed below.

Measurements from AMTAS^TM^ were excluded if the AMTAS^TM^ quality indicator indicated poor quality. A test for one subject, a retest for another subject, and both test and retest for a third subject were excluded for this reason. Measurements from the R-App were excluded if threshold results were below the floor- or ceiling-limit of the system. 17 thresholds (9 upon test and 8 upon retest) out of 1696 thresholds measured with the R-App were excluded from the data analysis due to these effects invalidating the thresholds found.

Furthermore, two subjects’ measurements with the R-App were also eliminated from the dataset due to technical issues with a cable. Additionally, 2 measurements of 3000 Hz and 6000 Hz respectively were not measured by the audiologist during manual audiometry.

## Results

When comparing pure-tone averages (PTAs) between manual audiometry and the user-operated methods, some differences exist. The largest difference was between manual audiometry and the R-App, where the R-App had eight ears more in the normal hearing category and 9 ears less in the slight impairment category. See Table 2 for the full comparison.

**Table 2:** PTAs across methods.

PTAs	Manual	R-App	AMTAS
≤ 25 (normal hearing)	78	86	82
26–40 (slight impairment)	26	17	20
41–60 (moderate impairment)	2	3	3
> 60 (severe impairment)	0	0	1
n	106	106	106

Comparison of pure-tone averages (PTAs) from manual audiometry, the R-App audiometry, and AMTAS audiometry. Only ears where PTA was available for all three tests are included.

### Accuracy of R-App smartphone user-operated audiometry

**Fig 2:**
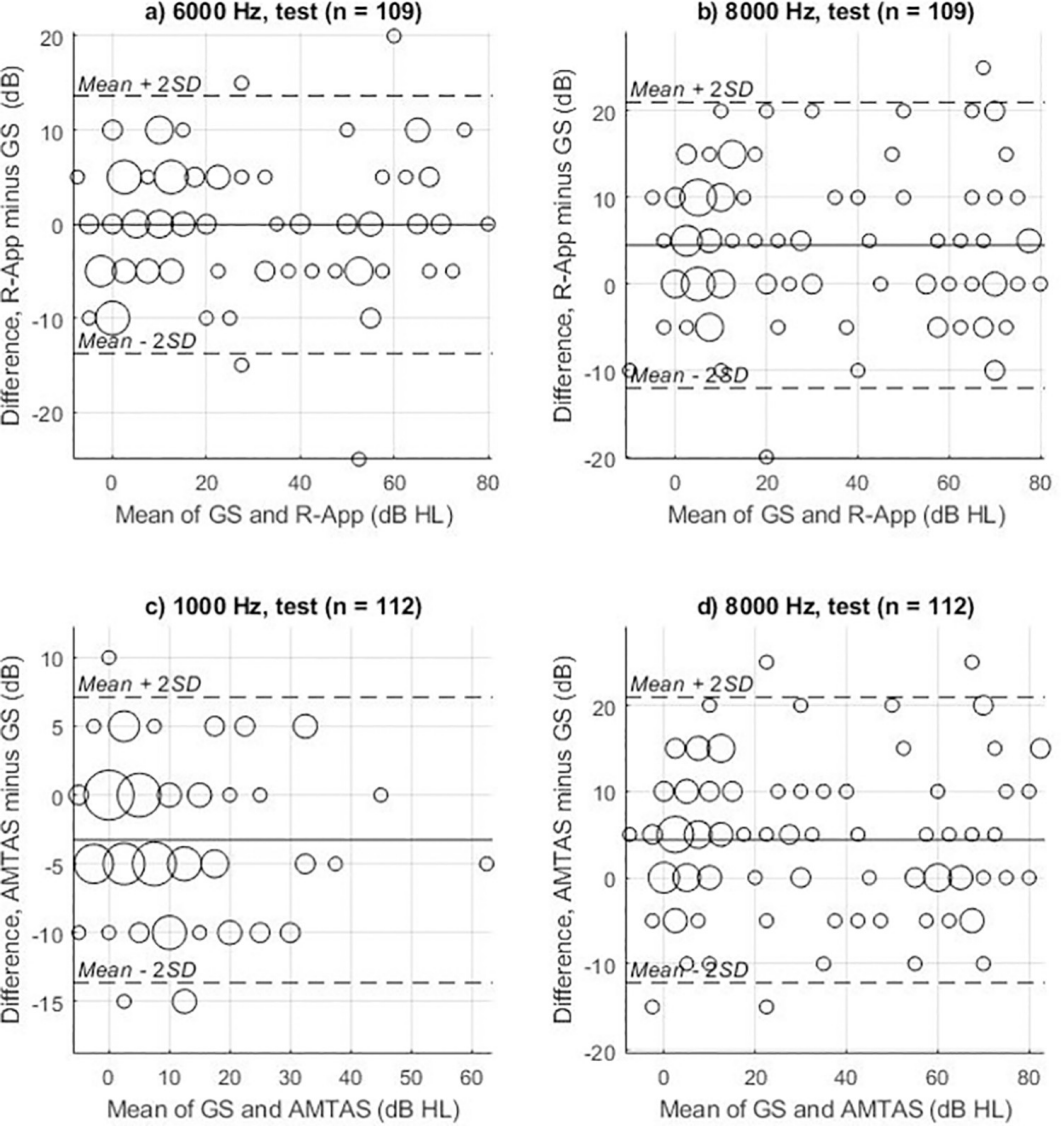
Bland-Altman plots for user-operated accuracy. Bland-Altman plots (difference plots) of the test thresholds obtained with different audiometry methods. a) and b) compare the R-App audiometry with the gold standard (GS) traditional manual audiometry, whereas c) and d) compare the AMTAS^TM^ audiometry with the manual audiometry. a) and c) show the frequencies with the lowest variability, whereas b) and d) show the frequencies with the highest variability (SD of absolute differences). The size of the circles indicates the number of threshold data-points at a particular mean threshold value. n = number of ears included in the comparison.

The number of threshold differences collected from the initial test was between 109 and 112, and from the retest it was 101–106. These threshold differences were analyzed to compare R-App and manual audiometry results for each air-conducted frequency. As shown in Table 3, the mean real difference between the R-App and manual audiometry ranged between -3.6 to 4.5 dB in initial test and -4.0 to 4.7 dB in retest, respectively. The SDs of the absolute differences ranged from 4.5 to 6.2 dB in the initial test and from 4.6 to 6.8 dB in the retest. The frequency with highest variability (highest SD of absolute difference) was 8000 Hz on both test and retest. As shown by the Bland-Altman plots of Fig 2A and 2B, threshold difference was not dependent on the mean threshold (hearing loss), i.e., no proportional bias was found when comparing the R-App–manual audiometry. Results of mixed-effects multilevel regression testing on the significance of R-App and manual audiometry differences are reported in Table 4.

**Table 3:** User-operated audiometry accuracy.

Frequency (Hz)	250	500	1000	2000	3000	4000	6000	8000
R-App test vs. manual audiometry: mean and SD of difference	-3.4 (7.9)	-3.6 (7.1)	-3.5(5.4)	-2.5(6.5)	-0.6 (6.8)	-0.1 (7.5)	0.0 (6.9)	4.5 (8.2)
R-App test vs. manual audiometry: mean and SD of absolute difference	6.9 (5.1)	5.9 (5.3)	4.4 (4.7)	5.1 (4.7)	5.0 (4.6)	5.2 (5.3)	5.2 (4.5)	7.1 (6.2)
*Number of ears*	*111*	*112*	*112*	*111*	*109*	*110*	*109*	*109*
R-App retest vs. manual audiometry: mean and SD of difference	-3.7 (6.7)	-4.0 (6.6)	-3.2 (5.7)	-3.0 (6.5)	-1.0 (6.3)	-0.4 (7.4)	0.3 (6.5)	4.7 (8.7)
R-App retest vs. manual audiometry: mean and SD of absolute difference	6.1 (4.6)	5.6 (5.3)	4.6 (4.7)	5.3 (4.8)	4.4 (4.6)	5.1 (5.3)	4.6 (4.6)	7.1 (6.8)
*Number of ears*	*106*	*106*	*106*	*105*	*102*	*105*	*101*	*105*
AMTAS^TM^ test vs. manual audiometry: mean and SD of difference	-0.9 (6.6)	-2.1 (7.3)	-3.3 (5.2)	-2.9 (7.0)	0.2 (6.0)	1.1 (7.6)	0.6 (6.2)	4.4 (8.3)
AMTAS^TM^ test vs. manual audiometry: mean and SD of absolute difference	4.9 (4.5)	5.4 (5.4)	4.7 (3.9)	5.4 (5.2)	3.7 (4.7)	5.0 (5.9)	4.3 (4.5)	7.1 (6.1)
*Number of ears*	*112*	*112*	*112*	*112*	*110*	*112*	*110*	*112*
AMTAS^TM^ retest vs. manual audiometry: mean and SD of difference	-1.0 (8.4)	-2.4 (6.9)	-4.1 (5.4)	-3.4 (6.8)	-1.2 (8.6)	-0.1 (7.6)	0.0 (7.2)	4.4 (9.6)
AMTAS^TM^ retest vs. manual audiometry: mean and SD of absolute difference	5.8 (6.2)	5.3 (5.0)	5.1 (4.5)	5.6 (5.2)	5.3 (6.9)	5.1 (5.6)	5.1 (5.0)	7.9 (6.9)
*Number of ears*	108	108	108	108	106	108	106	108

Mean difference and standard deviation (SD) in dB of real and absolute differences between manual audiometry & test, retest of R-App user-operated audiometry & test, retest of AMTAS^TM^. Manual audiometry is subtracted in the determination of differences.

**Table 4:** Statistical significance of user-operated audiometry accuracy.

Frequency	250	500	1000	2000	3000	4000	6000	8000
R-App test and manual audiometry. Fixed test effect coefficient and 95% confidence interval	-3.4*** (-5.2|-1.6)	-3.6*** (-5.0|-2.1)	-3.5*** (-4.8|-2.1)	-2.5** (-4.1|-0.9)	-0.6 (-2.6|1.3)	-0.1 (-1.8|1.6)	-0.1 (-2.1|1.9)	4.6*** (2.4|6.7)
Number of observations and number of groups	223 (56)	224 (56)	224 (56)	223 (56)	221 (56)	222 (56)	221 (56)	221 (56)
R-app retest and manual audiometry. Fixed test effect coefficient and 95% confidence interval	-3.7*** (-5.3|-2.1)	.4.0*** (-5.5|-2.5)	-3.2*** (-4.7|-1.6)	-3.0** (-4.7|-1.3)	-0.9 (-3.0|1.1)	-0.3 (-2.2|1.6)	0.2 (-1.7|2.1)	4.7*** (2.5|7.0)
Number of observations and number of groups	212 (53)	212 (53)	212 (53)	211 (53)	208 (53)	211 (53)	207 (53)	211 (53)
AMTAS^TM^ test and manual audiometry. Fixed test effect coefficient and 95% confidence interval	-1.0 (-2.6|0.7)	-2.1 (-3.7|-0.5)	-3.3*** (-4.8|-1.9)	-2.9** (-4.6|-1.1)	0.2 (-1.8|2.3)	1.1 (-0.7|2.8)	0.6 (-1.3|2.6)	4.4*** (2.2|6.5)
Number of observations and number of groups	228 (58)	228 (58)	228 (58)	228 (58)	226 (58)	228 (58)	226 (58)	228 (58)
AMTAS^TM^ retest and manual audiometry. Fixed test effect coefficient and 95% confidence interval	-1.1 (-3.0|0.8)	-2.4** (-4.0|-0.9)	-4.2*** (-5.7|-2.7)	-3.4*** (-5.2|-1.7)	-1.2 (-3.4|1.0)	-0.1 (-2.0|1.7)	-0.1 (-2.1|2.0)	4.4*** (2.1|6.7)
Number of observations and number of groups	220 (56)	220 (56)	220 (56)	220 (56)	218 (56)	220 (56)	218 (56)	220 (56)

Results of the mixed-effects multilevel regression testing for test and manual audiometry. The fixed test coefficient for 500 Hz AMTAS^TM^ and manual audiometry p-value was 0.011 but was not found to be significant after bonferroni-holm correction. Significant differences between tests are marked with the following:

* = p<0.05

** = p<0.01, and

*** = p<0.001.

### Accuracy of AMTAS^TM^ user-operated audiometry

A range of 110–112 threshold differences from the initial test and 106–108 threshold differences from the retest were analyzed to compare the AMTAS^TM^ and manual audiometry results for each air-conducted frequency. The mean real difference between AMTAS^TM^ and manual audiometry ranged between -3.3 to 4.4 dB in the initial test and -4.1 to 4.4 dB in the retest. SDs of absolute differences ranged between 3.9 to 6.1 dB in first test and 4.5 to 6.9 dB in the retest (Table 3). Results of mixed-effects multilevel regression testing on the significance of AMTAS^TM^ and manual audiometry differences are reported in Table 4.

The frequency with the highest variability on the AMTAS^TM^ was 8000 Hz in the first test and 3000 Hz on retest. Bland-Altman plots for the frequencies in the first test with the highest and lowest variability of the AMTAS^TM^—manual audiometry comparison showed no proportional bias (Fig 2C and 2D).

### R-App test-retest reliability

**Fig 3:**
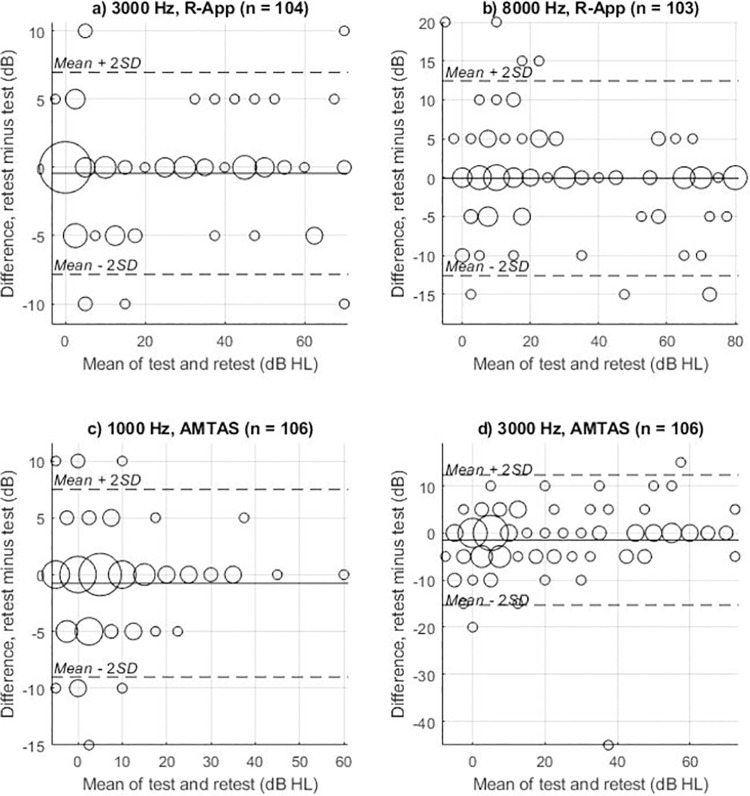
Bland-Altman plots for user-operated reliability. Bland-Altman plots (difference plots) of the test and retest thresholds. a) and b) are for the R-App audiometry, whereas c) and d) are for the AMTAS^TM^ audiometry. a) and c) show the frequencies with the lowest variability, whereas b) and d) show the frequencies with the highest variability (SD of absolute differences). The size of the circles indicates the number of threshold data-points at a particular mean threshold value. n = number of ears included in the comparison.

The R-App test-retest analysis provided 103–106 threshold difference measurements for each air-conducted frequency. As shown in Table 5, the mean real difference between test and retest thresholds was within ±1 dB, ranging from -0.6 dB (2000 Hz) to 0.4 dB (6000 Hz). The SDs of the absolute differences ranged from 3.1 to 5.0 dB, with 8000 Hz frequency having the highest SD. Results of mixed-effects multilevel regression testing on the significance of R-App test and retest differences are reported in Table 6. Bland-Altman plots for the frequencies with the highest and lowest variability of the test-retest comparisons showed no proportional bias (Fig 3A and 3B).

**Table 5:** User-operated audiometry reliability.

Frequency (Hz)	250	500	1000	2000	3000	4000	6000	8000
R-App mean and SD of difference	-0.4 (5.8)	-0.5 (4.2)	0.1 (4.1)	-0.6 (4.5)	-0.4 (3.7)	-0.3 (5.5)	0.4 (5.4)	-0.1 (6.3)
R-App mean and SD of absolute difference	3.6 (4.5)	2.0 (3.7)	2.1 (3.5)	2.5 (3.8)	2.1 (3.1)	3.7 (4.0)	3.3 (4.4)	3.8 (5.0)
*Number of ears*	105	106	106	104	104	103	103	103
AMTAS^TM^ mean and SD of difference	0.3 (6.4)	-0.1 (5.3)	-0.8 (4.1)	-0.5 (5.4)	-1.5 (6.9)	-1.5 (6.5)	-0.9 (5.2)	0.0 (7.2)
AMTAS^TM^ mean and SD of absolute difference	3.7 (5.2)	3.3 (4.2)	2.4 (3.5)	2.8 (4.7)	4.0 (5.8)	4.1 (5.2)	3.0 (4.3)	4.9 (5.2)
*Number of ears*	106	106	106	106	106	106	106	106

Test and retest mean difference and their standard deviation (SD) in dB of real and absolute differences of air-conducted thresholds at each tested frequency using R-App smartphone user-operated audiometry or AMTAS^TM^. Test is subtracted from retest in the determination of differences.

**Table 6:** Statistical significance of user-operated audiometry reliability.

Frequency (Hz)	250	500	1000	2000	3000	4000	6000	8000
R-App test-retest. Fixed test effect coefficient and 95% confidence interval	-0.4 (-2.0|1.3)	-0.5 (-1.8|0.8)	0.1 (-1.2|1.3)	-0.6 (-2.3|1.0)	-0.4 (-2.3|1.5)	-0.3 (-2.1|1.5)	0.3 (-1.6|2.2)	-0.2 (-2.3|2.0)
Number of observations and number of groups	211 (53)	212 (53)	212 (53)	210 (53)	210 (53)	209 (53)	208 (53)	208 (53)
AMTAS^TM^ test-retest. Fixed test effect coefficient and 95% confidence interval	0.2 (-1.5|2.0)	-0.1 (-1.7|1.5)	-0.8 (-2.1|0.6)	-0.5 (-2.3|1.2)	-1.5 (-3.6|0.6)	-1.4 (-3.3|0.4)	-0.9 (-2.8|1.1)	0.0 (-2.3|2.2)
Number of observations and number of groups	216 (55)	216 (55)	216 (55)	216 (55)	216 (55)	216 (55)	216 (55)	216 (55)

Results of the mixed-effects multilevel regression testing for test and retest. Significant differences between tests are marked with the following:

* = p<0.05

** = p<0.01, and

*** = p<0.001.

### AMTAS^TM^ test-retest reliability

A total of 106 threshold differences were included in the AMTAS^TM^ test-retest analysis for each tested frequency. As shown in Table 5, the mean real differences between test and retest were within ±1.5 dB, ranging from -1.5 to 0.3 dB. The SDs of the absolute differences ranged between 3.5 and 5.8 dB, with the frequency having the highest variability being 3000 Hz. One subject had a test-retest difference of > 40 dB on one ear at 3000 Hz, representing a clear outlier in the Bland-Altman plot in Fig 3D. However, the test quality indication given by AMTAS^TM^ for this subject’s test and retest were “good” and “fair” respectively, therefore the data from this subject were kept in the analysis. Had this subject been excluded, the SD of the absolute differences for 3000 Hz would have been lower (4.3 dB) and the frequency with the highest variability would have been 4000Hz (5.0 dB SD of absolute differences across 105 observations). Results of mixed-effects multilevel regression testing on the significance of AMTAS^TM^ test and retest differences are reported in Table 6. The Bland-Altman plots for the frequencies with the lowest and highest variability of all 106 observations are shown in Fig 3C and 3D.

### Measurement uncertainty

A combined standard uncertainty and an expanded measurement uncertainty for the measurements in this study was calculated using the formula in the international standard for audiometric test methods ISO 8253–1:2010 Annex A. The relevant input quantities for this calculation were; Determined hearing threshold level (L’HT), audiometric equipment (δeq), and transducers and their fitting (δtr). Although masking noise was presented during AMTAS^TM^ testing, masking noise was not included in the uncertainty calculation to make the uncertainty measurements the same for both user-operated methods. The combined standard uncertainty for frequencies up to and including 4000 Hz was 4.5 dB, and 6.4 dB above 4000 Hz. The expanded measurement uncertainty for frequencies up to and including 4000 Hz was 9 dB, and 13 dB above 4000 Hz.

## Discussion

This study compares two systems of user-operated audiometry in the same non-sound-treated environment for comparison with manual audiometry performed in a standard clinical setting within a sound treated room. To our knowledge it is the first study to use an Android smartphone-based app (R-App) calibrated with a commercial DAC and a pair of circum-aural RadioEar DD450 headphones, giving the capacity to detect air-conducted thresholds below 10 dB HL, in a non-sound-treated setting. In addition, this study provides new data on air-conducted thresholds with the AMTAS^TM^ user-operated audiometry in a non-sound-treated environment for comparison with data from the R-app as well as manual audiometry data from the same subjects tested in standard clinical conditions.

### Real and absolute threshold differences

In Tables 3 and 5, mean and SD of both real and absolute differences are presented. The two user-operated methods are compared to manual audiometry in Table 3, whereas the reliability of the user-operated methods with test-retest analysis is presented in Table 5. Both the R-App and AMTAS^TM^ accuracy measurements gave mean real differences close to zero (±1.2 dB) at 3000, 4000, and 6000 Hz, with some noticeable negative bias. The user-operated methods in general found significantly lower thresholds at 250, 500, 1000, and 2000 Hz except for 250 Hz for AMTAS^TM^ than manual audiometry as shown in Table 4. However, the systematic bias between AMTAS^TM^ and manual audiometry was only between –0.9 to –2.4 dB whereas the R-App and manual audiometry was between –3.4 to –4.0 dB, on 250 and 500 Hz in both test and retest data. Both user-operated methods showed a considerable bias towards higher thresholds (4.4 to 4.7 dB) at 8000 Hz, than manual audiometry. As shown in Table 4, the user-operated methods produced generally “better” (lower) thresholds than the manual audiometry for the lower frequencies and worse (higher) for the 8000 Hz frequency, even though the user-operated methods were used in a non-sound-treated room where ambient noise would be expected to impact the lower frequencies more than the higher frequencies. The DD450 also has excellent noise attenuation at the higher frequencies, making ambient occlusion an unlikely explanation to begin with. For the 250 and 500 Hz tones, 14 and 2 measurements respectively went above limits out of the 600 measurements made in the 10-minute duration. With such rare occurrence it is unlikely that the lower thresholds on the lower frequencies would be due to subjects reacting to ambient noise rather than stimuli.

Both SDs of real differences and SD of absolute differences fell within our calculated expanded measurement uncertainty (9dB up to and including 4000Hz, 13 dB above 4000Hz). That user-operated audiometry methods can achieve test-retest standard deviation within ISO standards in a non-sound-treated room is important knowledge for future attempts at implementing the technology, as scalability will be an important factor to keep hearing healthcare accessible in high-income countries and to make it accessible in lower- and middle-income countries.

### Accuracy

The Bland-Altman plots do not suggest the existence of any proportional bias for either the R-App or AMTAS^TM^. When conducting audiometric testing in the clinic, a variation in hearing threshold of 10 dB or less between two tests is usually considered acceptable [28, 29]. While this measure provides a useful reference for normal variation between tests, the exact way the variation is expressed may vary. Therefore, some published studies have used the SD of real differences while some have used absolute differences when examining the variation between user-operated and manual thresholds [19]. In the international standard for audiometric testing (ISO 8253–1:2010) an uncertainty model is presented for calculating accepted variation in audiometric measurements, based on potential sources that are known to affect test results. In our study, the accuracy analysis of the R-App (mean of real difference of 0.0–4.7 dB and SD of absolute difference of 4.5–6.8 dB) showed variation within our calculated range of uncertainty on all frequencies on both test and retest (Table 3). These results were comparable to the 0.1–5.0 dB mean real difference range and the SD of absolute difference of 3.5–5.0 dB range found in a previous meta-analysis on the validity of user-operated audiometry [30] and are in agreement with SD of real differences findings of 3.9–4.7 dB in a study evaluating a smartphone user-operated audiometry app [16].

In our comparison of AMTAS^TM^ and manual audiometry, SD of absolute differences ranged from 3.9 to 6.9 dB on test and retest. These results included outliers that could not be discredited as faulty, i.e., the quality prediction in AMTAS^TM^ was either found to be good or fair. An example of such an outlier is depicted in Fig 3D. In addition, our study found SDs of both absolute (3.9 to 6.9 dB) and real (5.2 to 9.6 dB) differences that were lower on most octave frequencies (250–6000 Hz), except at the 8000 Hz retest, when compared to SDs of both absolute (5.9 to 6.9 dB) and real (7.7 to 9.4 dB) differences in previous AMTAS^TM^ studies [19]. Eikelboom et al. [19] also found SDs of the absolute differences to be significantly greater (on the octave frequencies excluding 8000 Hz) than what was found by earlier reports on the accuracy of AMTAS^TM^ [17, 19]. These earlier reports showed SDs of absolute differences (3.2 to 4.4 dB, when excluding 8000 Hz) which were lower across most tested frequencies than those reported in this study (3.9 to 6.2 dB on octave frequencies excluding 8000 Hz) [17]. It may be that ambient noise explains the higher uncertainty as all previous AMTAS^TM^ studies were conducted in a sound-treated setting compared to the non-sound-treated consultation room used in this study. However, in the present study we have used the circum-aural headphone RadioEar DD450 that included sound attenuation. It has previously been demonstrated by Smull, Madsen [31] that circum-aural headphones such as RadioEar DD450 and Sennheiser HDA200 provides possibilities of accurate testing in a non-sound-treated room down to 10 dB HL in the low frequencies (125 to 250 Hz) and to –10 dB HL in the high frequencies > 2 kHz. We cannot see an increased variability and higher hearing thresholds at the low frequencies in both the R-app and the AMTAS^TM^ compared to the high frequencies (Table 3). Actually, as shown in Table 4, both R-app and AMTAS^TM^ thresholds are significantly lower in the low frequencies compared to manual audiometry Therefore, this study supports the previous results that accurate testing of hearing can be performed in a non-sound-treated room condition [31]. It has also been pointed out that sound treated rooms are not necessary for many hearing testing applications [32].

### Test-retest reliability

In this study, R-App test and retest SDs of real differences from air-conducted thresholds ranged between 3.7 and 6.3 dB, with SD of absolute differences ranging between 3.1 and 5.0 dB across all tested frequencies (Table 5). This variation is in line with previous studies that have reported SDs between 4–7 dB [17, 33]. Newer studies of test and retest of user-operated audiometry [34], and user-operated tablet-based audiometry [35] on normal hearing subjects also found similar ranges to this study, with a SD of real differences of 3.9–5.2 dB [34] and SD of absolute differences of 0.0–5.5 dB [35]. This gives further evidence regarding applicability and validity of the use of low-cost user-operated pure-tone audiometry apps that are developed and/or employed by researchers and caregivers with access to calibration equipment.

A recent study on test-retest of AMTAS^TM^ in a non-sound-treated setting found a mean SD of real differences of 3.7 dB with a difference of ≤5 dB in 93.5% of measurements [36]. However, only the 1000 Hz frequency was included in the test-retest analysis, lacking frequencies that are crucial when ambient occlusion is a concern for test validity. Earlier studies on AMTAS^TM^ found test-retest SD of absolute differences between 4.1–6.0 dB [19] and 2.8–4.7 dB [17], and SD of 5.4–12.2 dB [19] and 3.6–7.2 dB [17]. Within our study, we found similar variation (SD of absolute differences of 3.5 to 5.8 dB and SD of real differences of 4.1 to 7.2 dB) at the frequencies we tested. Meaning this study found better results in a non-sound-treated setting than previous studies in non-sound-treated settings, and similar results as previous studies in sound-treated settings.

### Study strengths and limitations

Audiometric examinations are made upon presentation of suspected hearing loss. With hearing loss becoming a more prevalent health concern, it is pertinent that new methods for determining hearing capacity that are readily accessible become available in the future, fit to the needs of many different contexts. It is important to note that the AMTAS^TM^ psychophysical test paradigm is available in a commercial audiometer whereas the R-app is not. The study proposes that it is possible to use mobile phones and a DAC with a psychophysical test paradigm for user-operated audiometry. If the test should be used for clinical purposes, it will require that the equipment should be approved for that purpose. However, the headphones used in the present study for the R-app and AMTAS^TM^ are approved for audiometry purposes.

Although this study included hearing impaired subjects, it also included many otologically normal subjects. This proved to be a strength of the study, as the Bland-Altman plots did not reveal any proportional bias, hence the quality of the results were not impacted by the subject’s ability to hear.

Ideally, evaluation of new test methods should be done by other researchers or clinicians not involved in the development of the methods. The present study evaluates the AMTAS^TM^ which has been developed by Margolis et al. [17]. However, the R-app was developed by the authors of the present study, and this introduces a risk of bias, as the R-app was also evaluated by the authors. It is, however, not considered to have a noticeable impact of the test results, but it is important to be aware of this fact.

One limitation of this study is that only the user-operated audiometries were counterbalanced for order effects. Always having manual audiometry come first meant that subjects that completed both manual and user-operated audiometry in one day might have become tired, and exhaustion would always carry over into the user-operated tests. Those test subjects were also never naïve listeners for the user-operated tests, as they would have been if full balancing had been done. A limitation of our study of the R-App included the inability to detect thresholds as low as -10 dB HL on all frequencies. As many test subjects had normal hearing according to PTA, we were not able to accurately detect all thresholds below the floor effect of the equipment on the mid frequencies. This was not an issue for the higher frequencies, as can be seen in Fig 2B and 2D. Another limitation of the R-App was a lack of detection when the subject reached unrealistic low (or high) dB HL. A limitation that can be quickly improved, as it can be overcome by having thresholds set by the calibration values and theoretical floor effect. Overcoming this limitation might present as a research opportunity in how to best handle test subjects that might be using the test wrong (if they are not simply deaf at the tested tone). A temporary lapse in concentration or a complete misunderstanding of the stimuli that should be listened for are likely cases where a test will experience this behavior from the subject. Different strategies could be implemented, i.e., giving the subject the option for playing the stimuli at a comfortable level to realign what they should listen for, or not giving the option but simply bring the test back to that level and let it continue.

The automated audiometry methods in the current study only use air conduction audiometry thresholds which limits the possibility of diagnosing conductive hearing loss. To diagnose a conductive hearing loss it is necessary to use a bone conductor, which has been tested with the AMTAS system [17, 37]. However, it is unlikely to find a conductive hearing loss if hearing thresholds in the low frequencies <1 kHz are <20 dBHL and when hearing thresholds in general are symmetrical. Therefore, it may be needed to use bone-conduction audiometry in case of large asymmetries and low frequency hearing loss in order to rule out a conductive component of the hearing loss.

## Conclusion

The accuracy of air-conducted thresholds of both R-App smartphone user-operated audiometry and AMTAS^TM^ were comparable to manual audiometry in terms of means and standard deviations. The test-retest reliability of both user-operated methods was very acceptable, giving variation within expected levels (based on ISO 8253–1:2010 Annex A) on all tested frequencies. Calibrated smartphone user-operated audiometry could potentially serve as a viable and cost-effective audiometric method for detection of air-conducted thresholds, and in contexts where pricing is less of a constraint the AMTAS^TM^ brings valuable quality indicators. Both user-operated methodologies gave reliable results when carried out in a non-sound-treated room.

## Supporting information

S1 Dataset Evaluation study data set. (XLSX)
